# Live imaging and biophysical modeling support a button-based mechanism of somatic homolog pairing in *Drosophila*

**DOI:** 10.7554/eLife.64412

**Published:** 2021-06-08

**Authors:** Myron Barber Child, Jack R Bateman, Amir Jahangiri, Armando Reimer, Nicholas C Lammers, Nica Sabouni, Diego Villamarin, Grace C McKenzie-Smith, Justine E Johnson, Daniel Jost, Hernan G Garcia

**Affiliations:** 1 Department of Molecular and Cell Biology, University of California, BerkeleyBerkeleyUnited States; 2 Department of Physics, University of California, BerkeleyBerkeleyUnited States; 3 Biology Department, Bowdoin CollegeBrunswickUnited States; 4 Univ Grenoble Alpes CNRS, Grenoble INP, TIMC-IMAGGrenobleFrance; 5 Biophysics Graduate Group, University of California, BerkeleyBerkeleyUnited States; 6 Université de Lyon, ENS de Lyon, Univ Claude Bernard, CNRS, Laboratory of Biology and Modeling of the CellLyonFrance; 7 Institute for Quantitative Biosciences-QB3, University of California, BerkeleyBerkeleyUnited States; Institut Curie, PSL Research University, CNRSFrance; Weizmann Institute of ScienceIsrael

**Keywords:** pairing, live imaging, button model, homolog, *Drosophila*, homologous chromosomes, *D. melanogaster*

## Abstract

Three-dimensional eukaryotic genome organization provides the structural basis for gene regulation. In *Drosophila melanogaster*, genome folding is characterized by somatic homolog pairing, where homologous chromosomes are intimately paired from end to end; however, how homologs identify one another and pair has remained mysterious. Recently, this process has been proposed to be driven by specifically interacting ‘buttons’ encoded along chromosomes. Here, we turned this hypothesis into a quantitative biophysical model to demonstrate that a button-based mechanism can lead to chromosome-wide pairing. We tested our model using live-imaging measurements of chromosomal loci tagged with the MS2 and PP7 nascent RNA labeling systems. We show solid agreement between model predictions and experiments in the pairing dynamics of individual homologous loci. Our results strongly support a button-based mechanism of somatic homolog pairing in *Drosophila* and provide a theoretical framework for revealing the molecular identity and regulation of buttons.

## Introduction

Eukaryotic genomes are highly organized within the three-dimensional volume of the nucleus, from the large scale of chromosome territories to the smaller-scale patterned folding of chromosomal segments called topologically associated domains (TADs) and the association of active and inactive chromatin into separate compartments (Szabo et al., 2019). Disruption of these organizational structures can have large consequences for gene expression and genome stability (Lupiáñez et al., 2015; Kragesteen et al., 2018; Despang et al., 2019; Rosin et al., 2019), emphasizing the importance of fully understanding the mechanisms underlying three-dimensional genome organization.

While many principles of genome organization are common among eukaryotes, differences have been noted between organisms and cell types. For example, in somatic cells in *Drosophila*, an additional layer of nuclear organization exists: homologous chromosomes are closely juxtaposed from end to end, a phenomenon known as somatic homolog pairing (Joyce et al., 2016; Stevens, 1908). While similar interchromosomal interactions occur transiently in somatic cells of other species and during early meiotic phases of most sexually reproducing eukaryotes, the widespread and stable pairing of homologous chromosomes in somatic cells of *Drosophila* appears to be unique to Dipteran flies (King et al., 2019; Joyce et al., 2016; McKee, 2004). Notably, the close juxtaposition of paired homologs can have a dramatic impact on gene expression through a process known as transvection, whereby regulatory elements on one chromosome influence chromatin and gene expression on a paired chromosome (Fukaya and Levine, 2017; Duncan, 2002). Although somatic homolog pairing was first described over 100 years ago (Stevens, 1908), the molecular mechanisms by which homologous chromosomes identify one another and pair have yet to be described.

**Figure 1.**
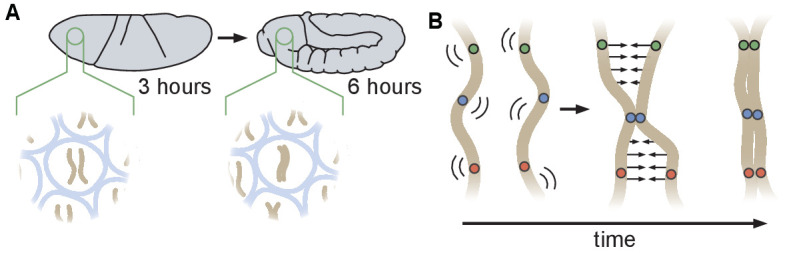
Schematic of homologous chromosome pairing in somatic cells in *D. melanogaster*. (**A**) Over the course of embryonic development, homologous chromosomes pair along their lengths. (**B**) Button model for homolog pairing in which each chromosome carries a series of sites that have affinity for the same site on its homologous chromosome.

During the early stages of *Drosophila* development, maternal and paternal genomes are initially separated and become paired as embryogenesis proceeds. Prior analyses of the initiation of somatic homolog pairing have relied primarily on DNA fluorescent in situ hybridization (DNA-FISH) to label homologous loci in fixed embryos, and have led to a model in which somatic homolog pairing slowly increases with developmental time through independent associations along the lengths of each chromosome arm (Figure 1A; Fung et al., 1998; Hiraoka et al., 1993; Gemkow et al., 1998). This model is further supported by recent studies that converged on a ‘button’ model for pairing, which hypothesizes that pairing is initiated at discrete sites along the length of each chromosome (Figure 1B; Viets et al., 2019; Rowley et al., 2019). However, the molecular nature of these hypothesized buttons is as yet unclear, nor is it clear whether this proposed model could lead to de novo pairing in the absence of some unknown active process that identifies and pairs homologous loci.

Here we turned the ‘button’ mechanism for somatic homolog pairing into a precise biophysical model that defines parameters for the activities of pairing buttons, informed by observations of pairing dynamics in living cells. Our simulations showed that chromosome-wide pairing can be established through random encounters between specifically interacting buttons that are dispersed across homologous chromosomes at various possible densities using a range of binding energies that are reasonable for protein–protein interactions. Importantly, we found that active processes are not necessary to explain pairing via our model, as all of the interactions necessary for stable pairing are initiated by reversible random encounters that are propagated chromosome-wide. We tested our model and constrained its free parameters by assessing its ability to predict pairing dynamics measured via live imaging. Our model successfully predicted that, once paired, homologous loci remain together in a highly stable state. Furthermore, the model also accurately predicted the dynamics of pairing through the early development of the embryo, as measured by the percentage of nuclei that become paired as development proceeds and by the dynamic interaction of individual loci as they transition from unpaired to paired states. In sum, through an interplay between theory and experiment aimed at probing molecular mechanisms, our analysis provides quantitative data that strongly support a button model as the underlying mechanism of somatic homolog pairing and establishes the conceptual infrastructure to uncover the molecular identity, functional underpinnings, and regulation of these buttons.

## Results

### Formalizing a button-based polymer model of homologous pairing

**Figure 2.**
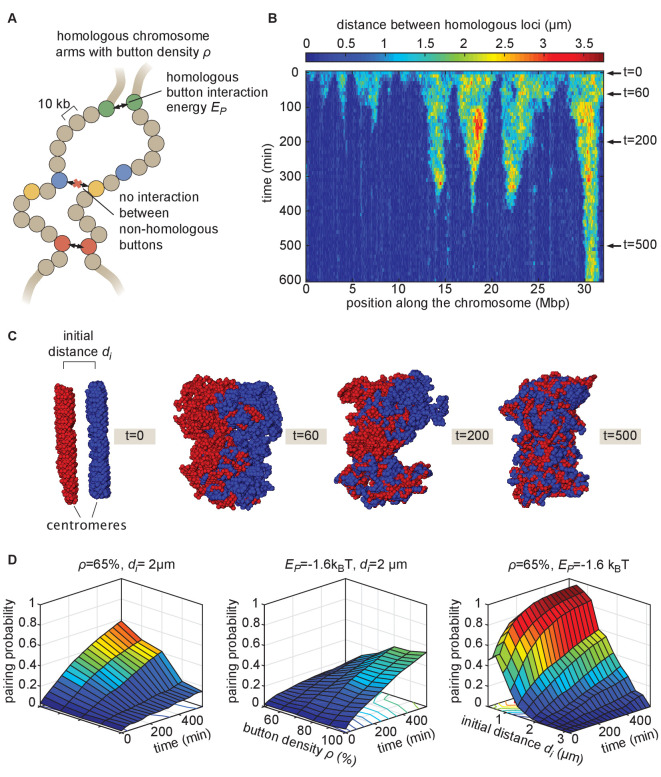
The homologous button model. (**A**) Pairing between homologous chromosomes is assumed to be driven by specific, short-range attractive interactions of strength $E_{p}$ between certain homologous regions, named buttons. Each 10-kb monomer in the simulation corresponds to one locus. (**B**) Kymograph of the time evolution of the distances between homologous regions predicted by the model in one representative simulated stochastic trajectory for a button density of ρ* = *65%, an interaction strength of $E_{p}$ = −1.6k_B_T, and an initial distance $d_{i}=1\mu m$. See Figure 2—figure supplement 1 for other examples for various $d_{i}$ values. (**C**) Snapshots of the pair of homologous chromosomes at various time points along the simulation in (**B**) (see also Figure 2—video 1 and 2). (**D**) Predicted average pairing probability between euchromatic homologous loci (considered as paired if their relative distance ≤1μm) as a function of time and of the strength of interaction $E_{p}$ (left), the button density ρ (center), and the initial distance $d_{i}$ between homologous chromosomes (right).

Prior studies have suggested that somatic homolog pairing in *Drosophila* may operate via a button mechanism between homologous loci (AlHaj Abed et al., 2019; Erceg et al., 2019; Fung et al., 1998; Gemkow et al., 1998; Rowley et al., 2019; Viets et al., 2019). In this model, discrete regions capable of pairing specifically with their corresponding homologous segments are interspersed throughout the chromosome. To quantitatively assess the feasibility of a button mechanism, we implemented a biophysical model of homologous pairing (Figure 2). Briefly, we modeled homologous chromosome arms as polymers whose dynamics are driven by short-range, attractive, specific interactions between homologous loci (buttons) to account for pairing (Materials and methods). These buttons are present at a density ρ along the chromosome and bind specifically to each other with an energy $E_{p}$. We included short-range, non-specific interactions among (peri)centromeric regions to account for the large-scale HP1-mediated clustering of centromeres (Materials and methods), which may also impact global genome organization inside nuclei (Rosin et al., 2018; Strom et al., 2017) and thus may affect pairing. As initial conditions for our simulations, we generated chromosome configurations with all centromeres at one pole of the nucleus (a ‘Rabl’ configuration; Figure 2—video 1 and 2), typical of early embryonic fly nuclei (Dernburg et al., 1996). To account for the potential steric hindrance of non-homologous chromosomes that could impede pairing, we simulated two pairs of homologous polymers. Note that other polymer models have previously been developed to study homologous pairing but mainly in a meiotic context: Nicodemi et al., 2008a; Nicodemi et al., 2008b proposed a generic model where homologous chromosomes are constrained to remain parallel and elongated and can interact via non-specific interactions, Penfold et al., 2012 investigated the role of centromeres and telomeres tethering in yeast on the inter-homolog distances but without accounting for any explicit pairing mechanisms, and Marshall and Fung, 2016 developed a glue-like model where homologous loci remain attached together when they first meet.

**Figure 2—figure supplement 1.**
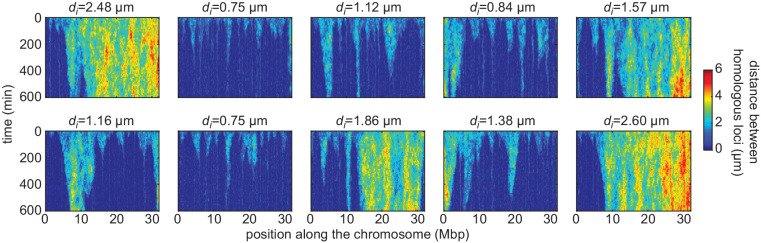
Simulated time evolution of distance between homologous loci. Examples of kymographs of the time evolution of the distances between homologous regions predicted by the model in several simulated stochastic trajectories with different initial distances *d_i_* for a button density of ρ* = *65% and an interaction strength of $E_{p}$ = −1.6k_B_T. Pairing operates via a ‘zippering’ mechanism: as one pair of homologous loci becomes stably paired, the pairing of adjacent buttons is facilitated, leading to the ‘spreading’ of pairing along the chromosome (flame-like patterns in the kymographs). For smaller initial distances, pairing is faster and more efficient.

**Figure 2—figure supplement 2.**
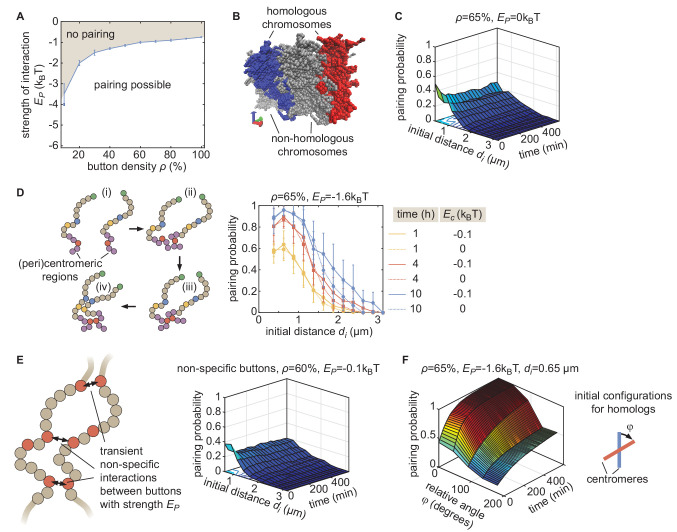
Properties of the button model. (**A**) Phase diagram of parameter ranges resulting in simulations compatible (white area) and incompatible (gray area) with pairing, starting from favorable initial homologous chromosome configurations (aligned chromosomes with $d_{i}<1$ µm, as in Figure 2C). (**B**) Representative simulation snapshot for ρ* = *65% and $E_{p}=-1.6$ kT. When homologous chromosomes (red and blue monomers) are initially distant, steric hindrance by other chromosomes (gray monomers) may prevent pairing. (**C**) Behavior in the absence of specific interaction ($E_{p}$ = 0) in the homologous button model. Predicted average pairing probability between euchromatic homologous loci (considered as paired if distance ≤1μm) as a function of time and the initial distance $d_{i}$ between homologous chromosomes. (**D**) Impact of HP1-mediated interaction between (peri)centromeric regions. (Left) Pairing between chromosomes that are initially distant (**i**) is facilitated and accelerated by the non-specific interactions between centromeric regions that lead to the clustering (**ii**) of HP1 regions. This clustering facilitates the pairing of homologous buttons located in centromeric regions (**iii**) and the subsequent pairing via the zippering process of nearby euchromatic regions (**iv**). (Right) Predicted average pairing probability between euchromatic homologous loci (considered as paired if distance ≤1μm) as a function of the initial distance $d_{i}$ between homologous chromosomes at 1 hr, 4 hr, and 10 hr for ρ* = *65% and $E_{p}$ = −1.6kT in the presence ($E_{c}=-0.1kT$, full lines) or absence ($E_{c}=0kT$, dashed lines) of interactions between (peri)centromeric regions. Even for large initial distances ($d_{i}\geq 2.5\mu m$), we observe a weak but significant amount of pairing in the euchromatic regions. These predictions are consistent with DNA-FISH experiments at various loci, suggesting an average higher pairing probability in heterochromatic loci during embryogenesis (see Figure 1 and Sup. Fig. 2 in Erceg et al., 2019). (**E**) The non-specific button model. To verify whether a non-specific button model can lead to global pairing, we relaxed the homologous model such that buttons may interact with any other buttons in the nucleus (left). In this model, the energy of a given configuration was described by.$$H=\sum_{chr}^{}\frac{k}{2}\sum_{i}^{}\left(1-cos\theta_{i,chr}\right)+\sum_{chr,chr^{\prime },i,j}^{}\left[E_{p}\delta_{chr,i;chr^{\prime },j}p_{chr,i}p_{chr^{\prime },j}+E_{c}\delta_{chr,i;chr^{\prime },j}C_{chr,i}C_{chr^{\prime },j}\right].$$ We varied ρ from 10 to 100% and $E_{p}$ from −0.025 to −4kT, one realization of which is shown here (right), without observing any global pairing of homologous chromosomes. Other parameters were as in the homologous button model (see Materials and methods of the main text). Contacts preferentially form between buttons belonging to the same chromosome, or more weakly, between buttons of different chromosomes but not necessarily between homologous loci. (**F**) Effect of the relative initial orientation between homologs. Predicted average pairing probability between euchromatic homologous loci (considered as paired if distance ≤1μm) as a function of time and the angle φ between the initial configurations of homologs (see inset), for an initial distance between the centers of mass of homologous chromosomes $d_{i}=0.65\mu m$. We see that lower relative angles lead to more efficient pairing, with Rabl-like configurations corresponding to 0°≤φ≤45°.

**Figure 2—figure supplement 3.**
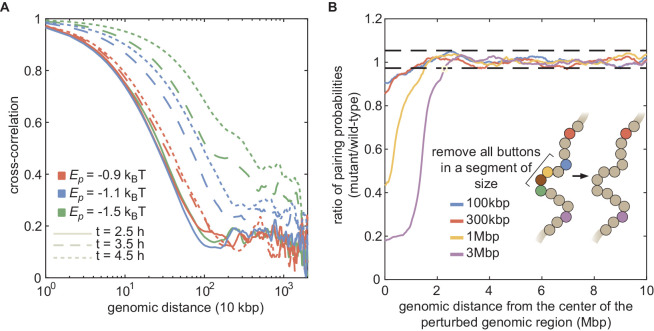
Large-scale correlations in pairing probabilities. (**A**) Cross-correlation of the simultaneous pairing status of two loci separated by a given genomic distance computed for different $E_{p}$ values (ρ = 80%) at different simulated developmental times. This cross-correlation mathematically corresponds to the correlation between a binary variable defining the pairing status at a given time *t* of a pair of homologous loci at position *i* (= 0 if unpaired and = 1 if paired) and the same variable but for another pair at position *j*, and averaged over all the possible couples of pairs (*i*, *j*) separated by the same genomic distance. Due to the polymeric nature of the chromosome, nearby loci (distant by less than 500kbp) remain significantly correlated even for weak $E_{p}$. However, while this correlation remains constant during development when pairing remains globally very low (red lines), the genomic range where loci tend to be correlated grows with developmental time as global pairing increases (blue and green lines). For example, for a scenario compatible with the experimental data ($E_{p}=-1.1kT$, blue lines), sites distant by more than 2 Mbp are highly correlated 2 hr after the beginning of the simulations (dotted line). (**B**) Investigation of the role of local perturbations of the button density on pairing efficiency. For parameters compatible with the experimental data (ρ = 80%, $E_{p}=-1.1kT$), we simulated mutant situations where all the buttons in a given chromosomal segment are removed and calculated their pairing dynamics. Full lines represent the ratio between the long-time (between 6 and 8 hr after the beginning of the simulations) local pairing probability in the mutant and the corresponding quantity in the wild-type situation (i.e., without button removal) as a function of the genomic distance to the central monomer of the perturbed chromosomal segment for different sizes of the perturbation (100 kbp, 300 kbp, 1 Mbp, 3 Mbp). The black dashed lines represent the limit of a 99% confidence interval characterizing the ratio of the local pairing probability between two simulation replicas of the wild-type case. We observed that the local perturbation propagates to distant sites. Indeed, the pairing probability is significantly reduced (ratio below the lowest black dashed line) up to approximately 800 kbp to 1 Mbp from the border of the mutated region.

**Figure 2—figure supplement 4.**
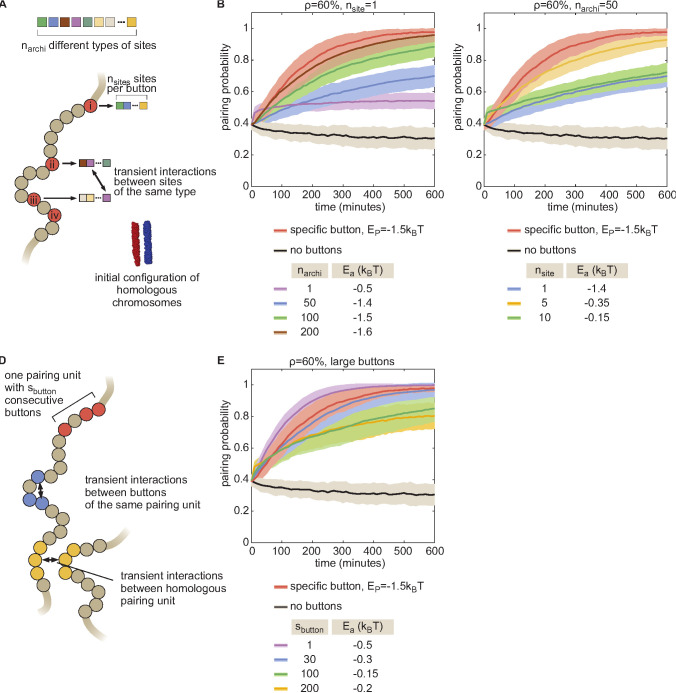
The combinatorial and large button models. (**A**) To investigate the possible nature of buttons, we developed a more general model in which buttons are made of *n_site_* binding sites for specific architectural proteins. Every site is occupied by one type of architectural proteins randomly chosen among *n_archi_* types. Two buttons may interact if they have common binding sites for some architectural proteins. The energy of a configuration is given by.$$H=\sum_{chr}^{}k\sum_{i}^{}\left(1-cos\theta_{i,chr}\right)+\sum_{chr,chr',i,j}^{}\left[E_{a}\delta_{chr,i;chr',j}m_{chr,i;chr',j}p_{chr,i}p_{chr',j}+E_{c}\delta_{chr,i;chr',j}C_{chr,i}C_{chr',j}\right]$$with parameters defined as in Equation S1, $m_{chr,i;chr',j}$ being the number of common binding sites between buttons (*chr,i*) and (*chr’,j*), and $E_{a}<0$ being the strength of interaction between binding sites bound to the same architectural proteins. Homologs share the same pattern of binding sites, that is $m_{chr,i;chr',i}=n_{site}$ if *chr* and *chr’* are homologous and if *i* is a button. For example, the case *n_site_* = 1, *n_archi_* = 1 ($m_{chr,i;chr',j}$ = 1 ,$E_{a}=E_{p}$) corresponds to the non-specific button model described in Figure 2—figure supplement 2E. To simplify and to avoid potential issues arising from periodic boundary conditions, we focused on favorable situations for pairing where homologs are initially aligned and close to each other (~640 nm between their respective centers of mass, see inset in **A**) and where the monomers evolve in a closed box (rigid wall conditions). Using this model, we first investigated how specific a button should be in order to lead to pairing. We fixed *n_site_* = 1 and varied *n_archi_* and $E_{a}$ for a button density of 60%. In these cases, *n_archi_* represents the number of button types. In (**B**), we plotted, for each *n_archi_*, the time evolution of the average pairing probability between homologous sites (paired if distance ≤1μm) for the $E_{a}$ value that leads to maximal pairing. As a point of comparison, we also plotted the corresponding curves in the absence of buttons (black line) and for the homologous button model investigated in the main text (red line) for the $E_{p}$ value (–1.5kT) consistent with experiments at the corresponding button density. We observed that as *n_archi_* increases (as the buttons become more specific) the pairing efficiency increases. The full specific model (red) becomes well approximated by our combinatorial button model for *n_archi_* > 200, i.e., when the number of buttons for one type is less than 10 per chromosome at 10kbp resolution. This suggests that pairing needs a significant degree of specificity via a large number of button types but each button type may be present in a small amount. However, we consider it unlikely that there are enough different architectural proteins in *Drosophila* to reach such single-site specificity. A possibility to increase specificity from a small number of proteins is to allow more than one binding site per button (*n_site_* > 1). The number of different buttons is then (*n_site_*)!/[(*n_archi_-n_site_*)!(*n_site_*)!]. In (**C**), we fixed *n_archi_* = 50, an upper maximal number of architectural proteins in flies, and varied *n_site_*. For each *n_site_*, we plotted the pairing probability for the $E_{a}$ value that leads to maximal pairing. We observed that there exists an optimal number of sites (here ~5) for which the pairing is close to the one obtained with the homologous button model. This corresponds to a value that leads to a large diversity of buttons while maintaining a low number of spurious interactions between non-homologous buttons, which is of the order of *n_site_*/*n_archi_*~0.1. (**D–E**) In the main text, we assumed that the size of a specific button corresponded to one monomer in our simulations (10 kbp). Recently, Viets et al., 2019 suggested that homologous TADs (average size ~100 kbp [Haddad et al., 2017]) may be the basic units of pairing. (**D**) To test this hypothesis, we developed a variant model that assumed that one pairing unit is composed by $s_{button}$ consecutive buttons along the genome. These buttons can interact specifically with each other and with their homologs at an interaction energy $E_{a}$. For example, the situation depicted in the main text corresponds to $s_{button}=1$. Formally this model is equivalent to the combinatorial model with $n_{site}$ = 1 and with *n_archi_* = (total number of buttons)/$s_{button}$ where buttons of the same type were placed consecutively along the chain (instead of randomly in the combinatorial model). As in (**A–C**), we simulated favorable situations where homologs are initially aligned and close to each other and where the monomers evolve in a closed box, using the same Hamiltonian as the combinatorial model. (**E**) Time evolution of the average pairing probability between homologous sites (paired if distance ≤1μm) for different $s_{button}$ values and the $E_{a}$ value that leads to maximal pairing for a fixed global button density of 60%. We observed that the level of pairing obtained for the full specific button model at an interaction strength compatible with experiments (red) can only be achieved if $s_{button}$ is smaller than ~40–50 buttons. This would correspond to pairing units of maximal size of about 750 kbp. For larger units, *cis* interactions (between buttons of the same unit on the same chromosome) dominate over *trans* interactions (between homologous buttons of the same unit) leading to less efficient pairing. TADs, that would correspond to $s_{button}$~ 5–15, are therefore possible pairing units compatible with pairing.

**Figure 2—video 1.** Polymer simulations of homologous pairing. Example of a 4-hr numerical simulation of the homologous button model (ρ = 60%, $E_{p}$ = −1.6 k_B_T) with frames taken every 30 s. The movie focuses on one pair of homologs (red and blue polymers). Orange and cyan parts of these chains represent their (peri)centromeric regions. Surrounding transparent light gray chains represent the periodic boundary images of the simulated chains. The scale bar is 1 µm. Homologous loci in closed contact (distance < 200 nm) are colored in green. Initially, chromosomes are randomly placed in a Rabl-like configuration. Then, they decompact and dynamically evolve in a crowded environment. First pairing events are diffusion-driven, followed by a spreading of pairing to nearest buttons via a zippering effect.

**Figure 2—video 2.** Polymer simulations of homologous pairing. Same as in Figure 2—video 1 but taken from a different simulation run. The two pairs of homologs are highlighted (red/blue for one pair; purple/dark blue). Orange/cyan and pink/light blue parts of these chains represent their (peri)centromeric regions. Surrounding transparent light gray chains represent the periodic boundary images of the simulated chains. The scale bar is 1 µm. Homologous loci in closed contact (distance < 200 nm) are colored in green.

When we monitored the distances between homologous loci in our simulations as a function of time (Figure 2B, Figure 2—figure supplement 1), qualitatively we observed that this thermodynamic model can lead to the time-progressive pairing of homologous chromosomes (Figure 2B) and the gradual intermingling of the two homologous chromosome territories (Figure 2C). Pairing in our simulations operates via a stochastic zippering process: once random fluctuations lead to the pairing of one pair of homologous loci, the pairing of nearest-neighbor buttons is facilitated along the lengths of the homologous chromosomes in a zipper-like manner (Figure 2B). Full chromosome-wide pairing results from the progression of many zippers that ‘fire’ at random positions and times along the chromosome, as also previously predicted for meiotic homologous pairing (Marshall and Fung, 2016).

We systematically investigated the roles of button density along the genome ρ, of the strength of the pairing interaction $E_{p}$, and of the initial distance between homologous chromosomes $d_{i}$ in dictating pairing dynamics (Figure 2D). For a given density, there is a critical value of $E_{p}$ below which no large-scale pairing event occurs independently of the initial conditions (Figure 2—figure supplement 2A) since pairing imposes a huge entropic cost for the polymers and thus requires a sufficient amount of energy to be stabilized. Beyond this critical point, higher strengths of interactions and higher button densities lead to faster and stronger pairing (Figure 2D, left, center). We also find that the non-specific interactions among (peri)centromeric regions included in our model facilitate pairing, but that such interactions are not strictly necessary (Figure 2—figure supplement 2D).

The initial spatial organization of chromosomes also strongly impacts pairing efficiency. When homologous chromosomes are initially far apart, pairing is dramatically slowed and impaired (Figure 2D, right) due to the presence of the other simulated chromosomes between them (Figure 2—figure supplement 2B). We also observed that our initial chromosome configurations corresponding to a Rabl-like organization (with all centromeres at one pole of the nucleus) promotes pairing by allowing homologous buttons to start roughly aligned (Marshall and Fung, 2016; Penfold et al., 2012; Nicodemi et al., 2008b; Figure 2—figure supplement 2F). Taken together, these systematic analyses of model parameters support the view that the homologous button model is compatible with pairing.

As an alternative model, we asked whether buttons that interact non-specifically could also explain somatic pairing. We simulated the dynamics of polymers having such non-specific buttons and never observed significant chromosome-wide pairing (Figure 2—figure supplement 2C,E). These results are complementary to previous works in which we showed that the weak, non-specific interactions between epigenomic domains that drive TAD and compartment formation in *Drosophila* (Ghosh and Jost, 2018; Jost et al., 2014) cannot establish and maintain stable pairing by themselves (Pal et al., 2019). Thus, in addition to button density, interaction strength, and initial organization of chromosomes, a key mechanism for pairing is the specificity of preferential interactions between homologous regions.

### Live imaging reveals homologous pairing dynamics

**Figure 3.**
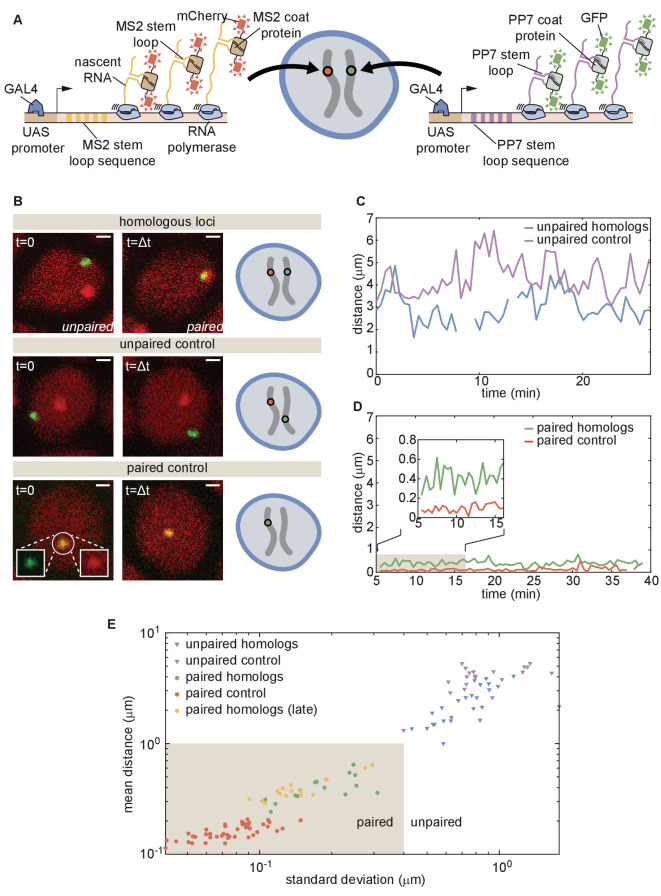
Live imaging of chromosomal loci provides dynamic single-locus spatiotemporal information about somatic homolog pairing. (**A**) Schematic of the MS2 and PP7 nascent mRNA labeling scheme for live imaging of homologous loci. Expression of the stem loops is driven by UAS under the control of a GAL4 driver. (**B**) Snapshots at two time points from homologous chromosomal loci with one allele tagged with MS2 and one allele tagged with PP7 (top), negative controls consisting of non-homologous loci labeled with MS2 and PP7 (middle), and positive controls corresponding to a single reporter containing interlaced MS2 and PP7 stem loops on the same chromosome (bottom). Scale bars represent 1 µm. See also Figure 3—videos 1, 2, 3, 4. (**C**) Representative traces of the dynamics of the distance between imaged loci for unpaired homologous loci and the negative control showing how both loci pairs have comparable distance dynamics. (**D**) Representative traces of the dynamics of the distance between imaged loci for paired homologous loci and the positive control demonstrating how the distance between paired loci is systematically higher than the control. (**E**) Mean and standard deviation (SD) of the distance between reporter transgenes, where each data point represents a measurement over the length of time that the loci were imaged (ranging from approximately 10–50 min, depending on the duration of the movie and the length of time that a nucleus remained in the field of view, see Figure 3—figure supplement 1). The shaded region indicates the criterion used to define whether homologs are paired (mean distance < 1.0 µm, SD < 0.4 µm) based on the distribution of points where homologs were qualitatively assessed as paired (yellow and green points).

The button model in Figure 2 makes precise predictions about pairing dynamics at single loci along the chromosome. To inform the parameters of the model and to test its predictions, it is necessary to measure pairing dynamics in real time at individual loci of a living embryo. To do so, we employed the MS2/MCP (Bertrand et al., 1998) and PP7/PCP (Chao et al., 2008) systems for labeling nascent transcripts. Here, each locus contains MS2 or PP7 loops that can be visualized with distinct colors in living embryos (Fukaya et al., 2016; Lim et al., 2018; Chen et al., 2018; Garcia et al., 2013). Specifically, we designed transgenes encoding MS2 or PP7 loops under the control of UAS (Brand and Perrimon, 1993) and integrated them at equivalent positions on homologous chromosomes (Figure 3A). Activation of transcription with GAL4 creates nascent transcripts encoding the MS2 or PP7 stem loops, each of which can be directly visualized by maternally providing fluorescently labeled MCP (MCP-mCherry) or PCP (PCP-GFP) in the embryo. The accumulation of fluorescent molecules on nascent transcripts was detected via laser-scanning confocal microscopy, providing relative three-dimensional positions of actively transcribing chromosomal loci in living *Drosophila* embryos (Lim et al., 2018; Chen et al., 2018).

**Figure 3—figure supplement 1.**
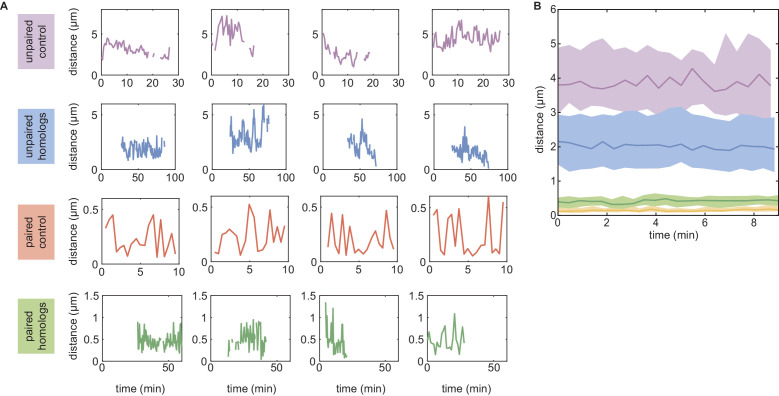
Experimental dynamics of inter-homolog distances used in Figure 3. (**A**) Examples of individual traces in the unpaired (first row) and paired (third row) control cases, and when homologs are unpaired (second row) or paired (fourth row) for locus 38F. For the unpaired control, we monitored 21 trajectories of ~27 min in duration. For the paired control, 44 trajectories of ~10 min were imaged. For the unpaired homologs, 30 trajectories of 20–70 min were acquired. Finally, for paired homologs, 25 trajectories of 7–50 min were measured. (**B**) For each of these four categories, we computed the median (full lines) trajectory over ~10 min. The corresponding interquartile ranges are shown as shaded areas. Monitored trajectories longer than 10 min were divided into several 10 min long trajectories to enrich the statistics.

**Figure 3—figure supplement 2.**
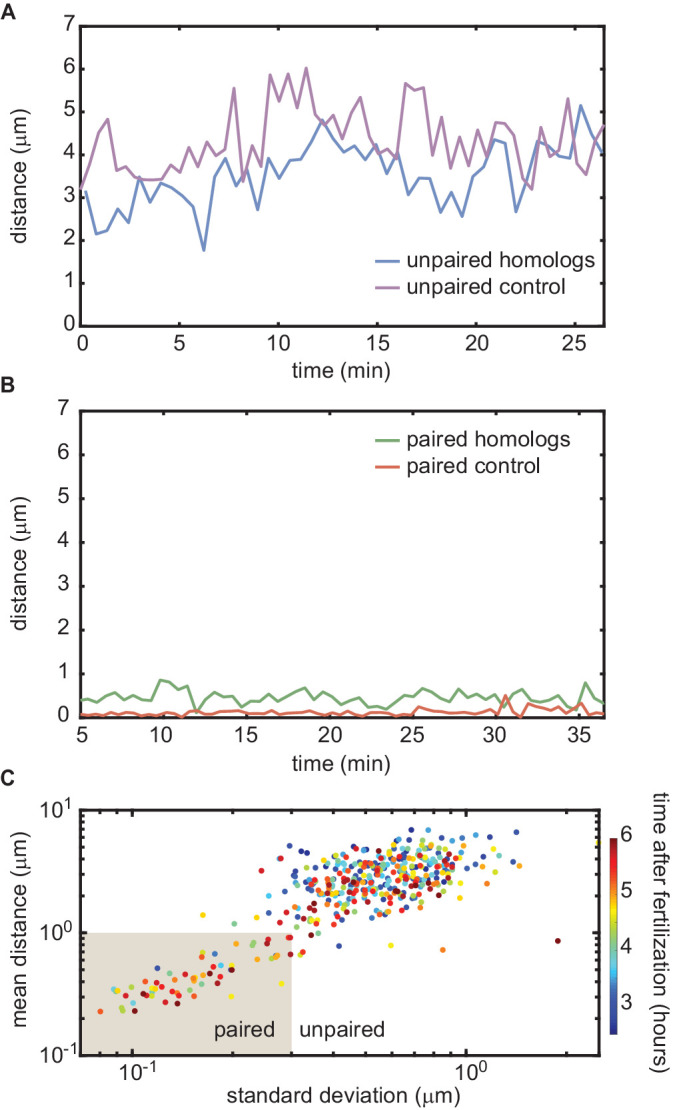
Homologous chromosome reporters inserted at the 53F genomic location. (**A**) Representative traces of the dynamics of the distance between imaged loci for unpaired homologous loci and the negative control. (**B**) Representative traces of the dynamics of the distance between imaged loci for paired homologous loci and the positive control. The traces in (**A**) and (**B**) are comparable to those measured at the 38F genomic location shown in Figure 3C,D. (**C**) Mean vs. standard deviation of the separation of each pair of homologous loci imaged in a single embryo over 6 hr of development (analyses of three embryos are combined). Each data point represents a 10 min window.

**Figure 3—video 1.** Representative confocal movie of a live *Drosophila* embryo (cell cycle 14 to gastrulation) in which MS2 and PP7 loops are integrated at equivalent positions on homologous chromosomes. Examples of nuclei are highlighted whose loci display characteristic dynamics, including loci that do not pair (‘Unpaired’), loci that are already paired (‘Paired’), and loci that are observed transitioning from the unpaired to the paired state (‘Pairing’). Image stacks were taken roughly every 30 s and max-projected for 2D viewing.

**Figure 3—video 2.** Representative confocal movie of a live *Drosophila* embryo (roughly 4.5 hr old) in which MS2 and PP7 loops are integrated at equivalent positions on homologous chromosomes. A greater proportion of nuclei show paired homologs relative to the earlier time point represented in Figure 3—video 1. Image stacks were taken roughly every 30 s and max-projected for 2D viewing.

**Figure 3—video 3.** Representative confocal movie of a live *Drosophila* embryo (roughly 5.5 hr old) in which MS2 and PP7 loops are integrated at various positions on homologous chromosomes (MS2 at position 38F and PP7 at position 53F) where we expect no pairing between transgenes. Image stacks were taken roughly every 30 s and max-projected for 2D viewing.

**Figure 3—video 4.** Representative confocal movie of a live *Drosophila* embryo (cell cycle 14) in which MS2 and PP7 loops were interlaced in a single transgene on one chromosome at polytene position 38F to act as a positive control for pairing. Both GFP and mCherry are co-localized to the same locus in all transcriptional loci. Image stacks were taken roughly every 30 s and max-projected for 2D viewing.

We focused on embryos that had completed the maternal-to-zygotic transition and began to undergo gastrulation at approximately 2.5–5 hr after embryo fertilization, when pairing begins to increase substantially (Fung et al., 1998). We integrated transgenes into two genomic locations on chromosome two at polytene positions 38F and 53F, and analyzed embryos with MS2 and PP7 loops at the same positions on homologous chromosomes in order to monitor pairing (Figure 3B, top; Figure 3—video 1 and 2; Materials and methods). As a negative control, we imaged embryos in which loops were integrated at two different positions on homologous chromosomes (MS2 at position 38F and PP7 at position 53F), where we expect no pairing between transgenes (Figure 3B, middle; Figure 3—video 3). Finally, as a positive control for the spatial colocalization of MS2 and PP7 loops, we analyzed embryos where MS2 and PP7 loops were interlaced in a single transgene (Wu et al., 2014; Chen et al., 2018) on one chromosome at polytene position 38F (Figure 3B, bottom; Figure 3—video 4). For each case, we imaged multiple embryos for 30–60 min, and used custom MATLAB scripts to determine the relative 3D distances between chromosomal loci over time (Figure 3—figure supplement 1; Materials and Methods).

In embryos with both PP7 and MS2 transgenes integrated at polytene position 38F (Figure 3B, top), the majority of nuclei could be qualitatively classified into one of two categories. In ‘unpaired’ nuclei, homologous loci were typically separated by > 1 µm with large and rapid changes in inter-homolog distances (e.g. Figure 3C, blue), with a mean distance of 2.2 µm and standard deviation (SD) of 1.2 µm averaged over 30 nuclei. The measured mean distance between homologous loci was comparable within error, though systematically smaller, than the mean distance between loci in the negative control, where transgenes were integrated at non-homologous positions (Figure 3C, red, mean distance = 4.0 µm, SD = 1.3 µm, n = 21 nuclei). In contrast, in ‘paired’ nuclei, homologous loci remained consistently close to one another over time, with smaller dynamic changes in inter-homolog distance (Figure 3D, blue, mean distance = 0.4 µm, SD = 0.3 µm, n = 25 nuclei). Interestingly, while the diffraction-limited signals produced from homologous loci occasionally overlapped in paired nuclei, their average separation was systematically larger than that of the positive-control embryos carrying interlaced MS2 and PP7 loops (Figure 3D, red, mean distance = 0.2 µm, SD = 0.1 µm, n = 44 nuclei). This control measurement also constitutes a baseline for the experimental error of our quantification of inter-homolog distances (Chen et al., 2018). Our measurements thus confirmed previous observations of transgene pairing in the early embryo in which signals from paired loci maintained close association but did not completely coincide over time (Lim et al., 2018). Notably, of 38 nuclei qualitatively scored as having paired homologs, we never observed a transition back to the unpaired state over a combined imaging time of more than 8 hr. Embryos with PP7 and MS2 transgenes integrated in homologous chromosomes at polytene position 53F showed comparable dynamics of inter-homolog distances for nuclei in unpaired and paired states (Figure 3—figure supplement 2A,B). Thus, somatic homolog pairing is a highly stable state characterized by small dynamic changes in the distance between homologous loci.

Our assessment thus far has been based on a qualitative definition of pairing. In order to devise a more stringent quantitative definition of homologous pairing, we measured inter-transgene distances for homologous loci as well as for the unpaired and paired controls throughout gastrulation. We also included measurements from older embryos (~11–12 hr after fertilization) using the driver *R38A04-GAL4* (Jenett et al., 2012) to express the transgenes in epidermal cells, where pairing is expected to be widespread (Fung et al., 1998; Gemkow et al., 1998). We measured the mean and SD of the inter-transgene distance for each nucleus over ~10–50 min. From these data, we established a quantitative and dynamic definition of somatic homolog pairing based on a mean distance < 1.0 µm and a corresponding standard deviation < 0.4 µm (Figure 3E, shaded region). By this definition, we considered paired 100% of nuclei that we had qualitatively scored as such, but excluded all nuclei scored as unpaired. As expected, this definition also scored 100% (15/15) of the tracked nuclei from older embryos as paired. Data for paired nuclei from early versus late embryos were in close agreement (Figure 3E, yellow), suggesting that pairing observed in early embryos is representative of pairing during later stages of development.

**Figure 4.**
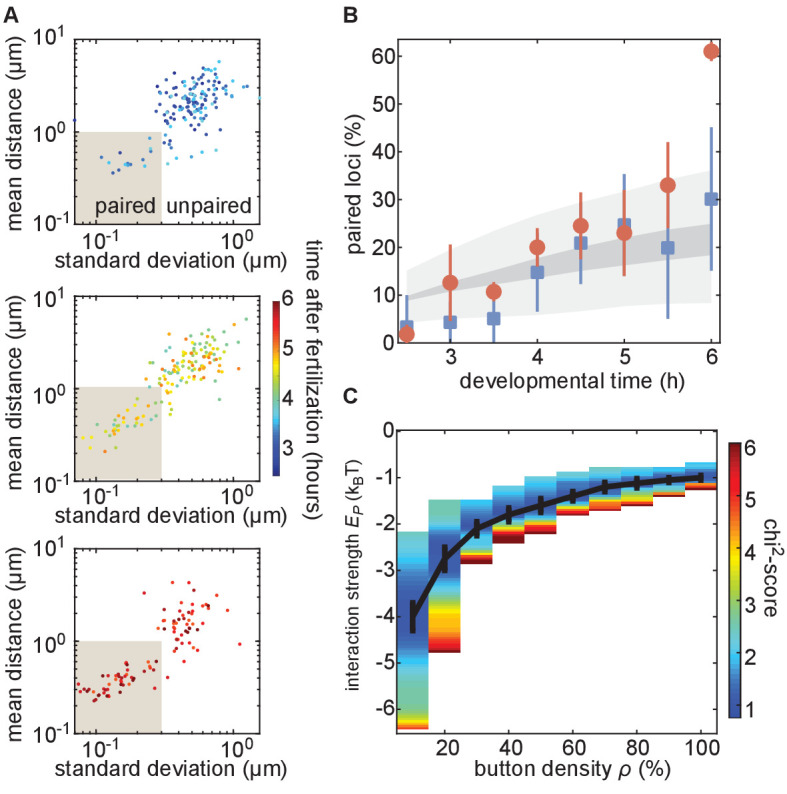
The homologous button model recapitulates the observed developmental dynamics of pairing. (**A**) Mean and SD of the separation of each pair of transgenes integrated at position 38F imaged in a single embryo over 6 h of development. Each data point represents a single nucleus over a 10-min time window, revealing the increase in the fraction of paired loci as development progresses. Data are separated into three plots for ease of visualization. (**B**) Nuclei from each time point were scored as “paired” if they fell within the shaded box in (**A**). Data were taken from three embryos each for transgenes at 38F (red) and 53F (blue) with error bars representing the standard error of the mean. For each button density ρ, we fitted the experimental pairing dynamics (Figure 4—figure supplement 2A). Gray shading provides the envelope of the best predictions obtained for each ρ (dark gray) and its SD (light gray). (**C**) Phase diagram representing, as a function of ρ, the value of $E_{p}$ (black line) that leads to the best fit between predicted and experimental developmental pairing dynamics. The predicted pairing strength is weaker than observed in the parameter space above the line, and stronger than observed below the line. Error bars represent the uncertainties on the value of $E_{p}$ that minimizes the chi^2^-score at a given ρ value.

We next analyzed the progression of pairing through the first 6 hr of development in single embryos carrying MS2 and PP7 transgenes in homologous chromosomes at positions 38F and 53F. To accomplish this goal, we collected data for short (~10 min) intervals every 30 min from 2.5 hr to 6 hr of development, and analyzed inter-homolog distances as outlined above. We then plotted the mean of this distance as a function of its SD for each nucleus analyzed at each time point to create a dynamic assessment of somatic homolog pairing over developmental time. As expected, we detected an overall decrease in mean inter-homolog distance and its SD as development progressed (Figure 4A, Figure 3—figure supplement 2C). To directly compare our analysis to prior studies, we binned nuclei into paired and unpaired states based on their mean and SD as defined in Figure 3E and plotted the percentage of paired nuclei at each developmental time point (Figure 4B). Consistent with previous literature (Fung et al., 1998; Gemkow et al., 1998), we observed a steady increase in the proportion of paired nuclei (Figure 4B); however, by our dynamical definition of pairing, the percentage of nuclei that are paired is systematically lower at most time points than results using DNA-FISH (Figure 4—figure supplement 1). This disagreement likely reflects differences between the classic, static definition of pairing based on overlapping DNA-FISH signals in the one snapshot accessible by fixed-tissue measurements as opposed to our dynamics-based definition, which demands that loci be paired over several consecutive frames. In sum, we have demonstrated that our system captures the progression of somatic homolog pairing over developmental time, making it possible to contrast theoretical predictions and experimental measurements.

**Figure 4—figure supplement 1.**
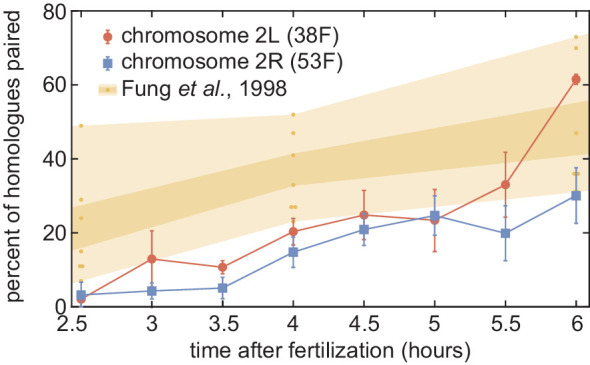
Comparison of our pairing data to previous results. Pairing dynamics measured by live imaging at two chromosomal loci (red and blue points) as presented in Figure 4B. The progression of pairing observed in fixed embryos using DNA-FISH was obtained from Fung et al., 1998; yellow points represent the pairing measured at seven euchromatic loci on chromosome arm 2L at three time points, and yellow shading represents standard error (dark) and range (light). Our data is consistent with, but systematically lower than, the range of pairing observed in the prior study.

**Figure 4—figure supplement 2.**
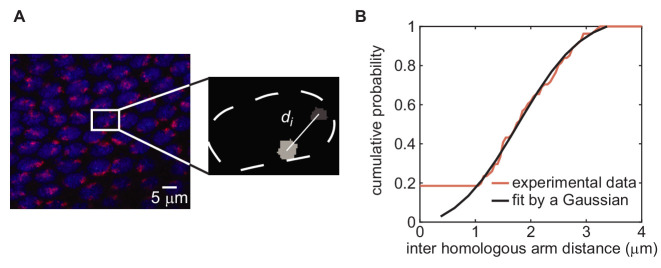
Establishing values for initial distance $d_{i}$ between homologous chromosomes via chromosome painting. (**A**) Chromosome painting of chromosome arm 2L (red), carrying transgene location 38F, in an embryo in early cycle 14. Nuclei (blue) are stained with DAPI. Inter-homolog distances were determined by segmenting painted regions in 3D using ImageJ and measuring center-to-center distances (inset; see Materials and methods for details). Chromosome arm 2R, carrying transgene location 53F, was sampled in the same field of cells using a different fluorescent tag (not shown). (**B**) Distribution of inter-homologous arm distances measured from 48 nuclei in the image in (**A**) (red curve). Measurements from chromosome arms 2L and 2R were completely overlapping and therefore were combined. The curve is fitted by a Gaussian distribution (black curve, mean = 1.9, SD = 0.9).

**Figure 4—figure supplement 3.**
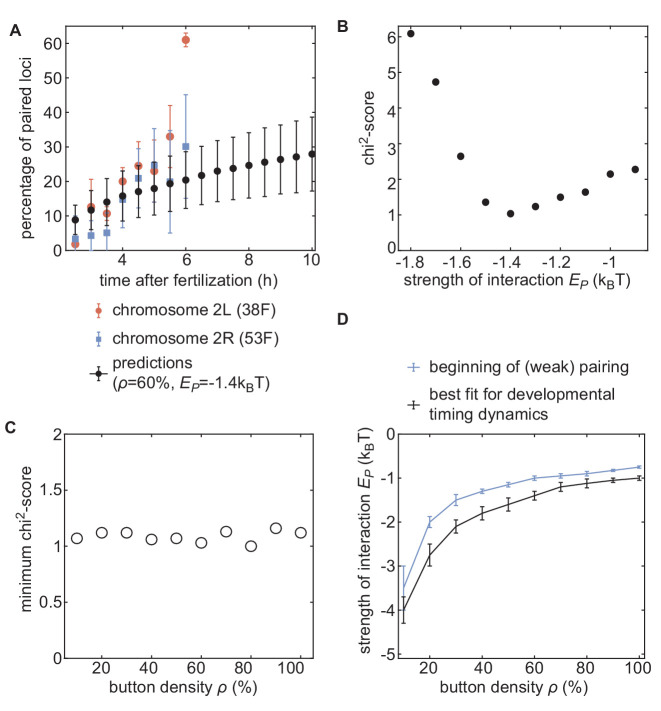
Inference of developmental pairing dynamics. (**A**) Predicted time evolution of the mean pairing dynamics computed over all buttons in the simulation (black dots) for a distribution of initial inter-homolog distances $d_{i}$ given in Figure 4A and for ρ= 60% and $E_{p}$ = −1.4 k_B_T. Error bars in simulated data represent SD of the pairing dynamics computed over all the buttons (n = 3200), while error bars in experimental data represent the standard error of the mean (n = 3 embryos for each chromosomal position). The model can recapitulate the experimentally measured pairing dynamics. (**B**) Evolution of the chi^2^-score as a function of $E_{p}$ for ρ= 60%. For some $E_{p}$ values, we performed two sets of independent simulations. (**C**) The values of the minimum chi^2^-score obtained for all the investigated button densities are very similar, suggesting an equivalent predicting power for every ρ. (**D**) Phase diagram representing, for each button density ρ, the value of $E_{p}$ (black line) that leads to the best fit between predicted and experimental developmental pairing dynamics. The blue line stands for the weak pairing transition defined in Figure 2—figure supplement 2A.

### Constraining the button model using dynamical measurements of pairing probability

Our button model predicts that the fraction of paired loci as a function of time depends on three parameters: the initial separation between homologous chromosomes *d_i_*, the density of buttons along the chromosome ρ, and the button–button interaction energy $E_{p}$ (Figure 2D). As an initial test of our model, and to constrain the values of its parameters, we sought to compare model predictions to experimental measurements of the fraction of paired loci over developmental time.

Due to the still unknown molecular identity of the buttons, it was impossible to directly measure the button density and the button–button interaction energy. However, the initial separation between chromosomes *d_i_* can be directly estimated using chromosome painting (Ried et al., 1998; Beliveau et al., 2012). To make this possible, we used Oligopaint probes (Beliveau et al., 2012) targeting chromosome arms 2L and 2R to perform chromosome painting on embryos ~ 130 min after fertilization, corresponding to the beginning of cell cycle 14 (Foe, 1989; Figure 4—figure supplement 2A). The resulting distribution of distances between homologous chromosome territories was well described by a simple Gaussian distribution for distances greater than 1 µm, roughly corresponding to the distance required to resolve two separate chromosome territories (Figure 4—figure supplement 2B, red line.)

We next investigated whether the button model quantitatively reproduced the pairing dynamics observed during development for reasonable values of the button density and the button–button interaction energy. We ran a series of simulations for various values of button density ρ (from 10% to 100%) and strength of interaction $E_{p}$ (from −0.5k_B_T to −5k_B_T) starting from values for the initial distance between homologous chromosomes *d_i_* drawn from the inferred Gaussian distribution from our Oligopaint measurements (Figure 4—figure supplement 2B, black line). For each parameter set, we monitored pairing dynamics as a function of developmental time and computed the average probability for a locus to be paired (Figure 4—figure supplement 3A, black points) using the same criterion as in Figure 3E. By minimizing a chi^2^-score (Figure 4—figure supplement 3B) between the predictions and the experimental pairing probability (Materials and methods), we inferred, for each button density, the strength of interaction that best fits the data (Figure 4C). Interestingly, the goodness of fit was mainly independent of button density (Figure 4—figure supplement 3C): denser buttons require less strength of interaction to reach the same best fit (black line in Figure 4C).

The inferred developmental dynamics quantitatively recapitulated the experimental observations for both investigated loci at the majority of time points analyzed (Figure 4B) for any choice of parameters given by the curve in Figure 4C. At our initial time point of 2.5 hr, we predict pairing to be slightly higher than observed for position 38F. This disagreement could reflect an underestimate of the initial distance *d_i_* in our simulations at distances less than 1 µm, which corresponds to the resolution limit of our Oligopaint-based measurements (Figure 4—figure supplement 2B), or that homolog pairing is not yet stable early in *Drosophila* development (Lim et al., 2018). We also find that our simulations did not predict the large increase in pairing observed for 38F between 5.5 hr and 6 hr (Figure 4B), which may be a consequence of the proximity of 38F to the highly paired histone locus body (Hiraoka et al., 1993; Fung et al., 1998). In sum, the button model recapitulates the observed average pairing dynamics for a wide range of possible button densities coupled with interaction energies that are consistent with protein-DNA interactions.

### Parameter-free prediction of individual pairing dynamics

The fit of our button model to the fraction of paired loci during development in living embryos (Figure 4B) revealed a dependency between the interaction strength $E_{p}$ and the buttondensity ρ (Figure 4C). As a critical test of the model’s predictive power, we sought to go beyond averaged pairing dynamics and used the model to compute the pairing dynamics of individual loci. As can be seen qualitatively in the kymographs predicted by the model (Figure 2B, Figure 2—figure supplement 1), pairing spreads rapidly (within tens of minutes) from the buttons that constitute the initial points of contact along the chromosome. As a result, the button model predicts that homologous loci undergo a rapid transition to the paired state as the zippering mechanism of pairing progression moves across the chromosome.

**Figure 5.**
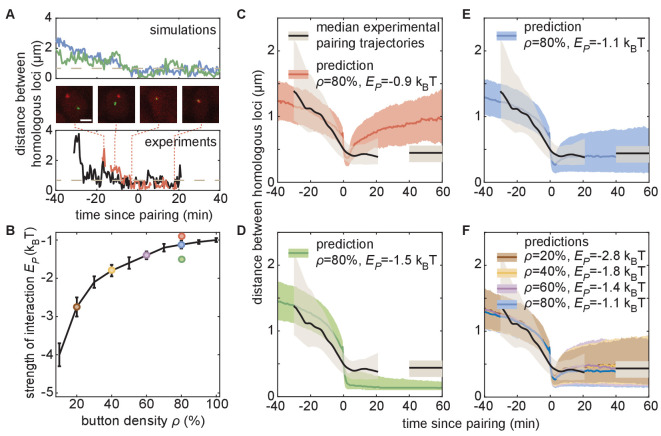
The homologous button model predicts individual pairing dynamics. (**A**) Examples of simulated (top) and experimental (bottom) pairing trajectories showing rapid transitions from the unpaired to the paired state. Simulations were carried out using ρ *=* 50% and $E_{p}=-1.75k_{B}T$. See also Figure 5—figure supplement 1. Scale bar is 2 μm. Dotted line in each graph represents our distance threshold for aligning pairing traces defined as the time point where the inter-homolog distance decreases below 0.65 µm for at least 4 min. (**B**) Parameter range inferred from pairing probability dynamics in Figure 4 (black line), and parameters used for the simulations in (**C–F**) (color points). (**C–F**) Median pairing dynamics obtained from individual pairing trajectories detected during our experiments (black lines) and simulations (colored lines). Traces are centered at the time of pairing (time = 0) as in (**A**). The long-term, experimentally measured inter-homolog distance is plotted as a straight black line at the right of each panel. The interquartile range of the distributions of distances between homologous loci are indicated by the shaded regions (n = 14 nuclei for experiments; n > 10,000 traces for simulations). Note that the experimental data were processed (Figure 5—figure supplement 1C) to smooth out the effect of small statistics (Figure 5—figure supplement 2C).

To quantify the predicted pairing dynamics of homologous loci, we collected single-locus traces containing individual pairing events from our simulations (Figure 5A, top), which we defined as traces in which the inter-homolog distance drops below 0.65 µm for at least 4 min (Materials and methods). For traces corresponding to each set of simulations with various values of ρ and $E_{p}$ (Figure 5B), we calculated the median dynamics of inter-homolog distances around the pairing event. Across many values of ρ and $E_{p}$, the medians of the predicted trajectories leading up to the pairing event were very similar, with inter-homolog distances decreasing rapidly from 1 to 2 µm to below 0.65 µm at an accelerating rate over the course of 10–20 min (Figure 5C–F). However, we do observe subtle differences in this pre-pairing stage: for a given button density, a stronger interaction energy $E_{p}$ leads to a faster approach of the homologs (Figure 5—figure supplement 2A). The nearly independence of this first period of the pairing dynamics with respect to ρ and $E_{p}$ suggests that the initial approach of homologous loci is mainly diffusion limited, while there is a slight acceleration of pairing for stronger interaction energies due to an enhanced zippering effect (Figure 5—figure supplement 2B).

**Figure 5—figure supplement 1.**
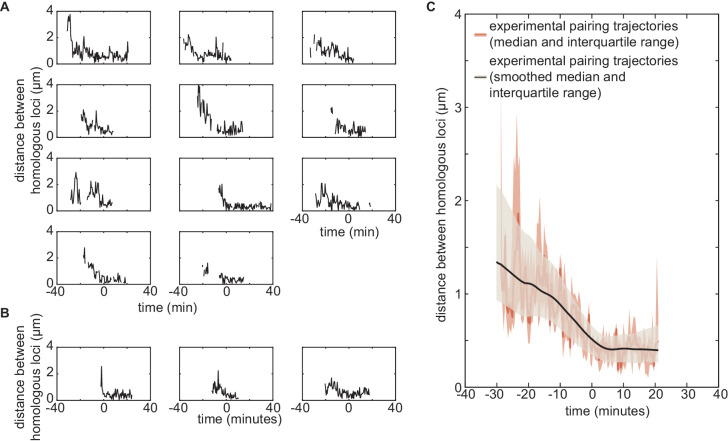
Experimental individual pairing dynamics. (**A,B**) Fast single-locus pairing dynamics illustrated by individual pairing traces detected for the experiments on locus 38F (**A**, n = 11) and locus 53F (**B**, n = 3) centered at the time of pairing (time = 0) defined as the time point where the inter-homolog distance decreases below 0.65 µm for at least 4 min. (**C**) From the 14 individual pairing trajectories observed experimentally, we estimated at each time point the median (dark red full line) and the interquartile range (light red shaded area) of the distribution of distances between homologous loci. The corresponding smoothed median is plotted in black, and the interquartile range (as in Figure 5 of the main text) is given by the gray shaded area. Smoothing was performed using the *smooth* function of Matlab (method = *lowess*, span = *40*). In Figure 5—figure supplement 2C, we demonstrate the effect of very low statistics and of smoothing on the overall shape of the pairing dynamics using simulated data.

**Figure 5—figure supplement 2.**
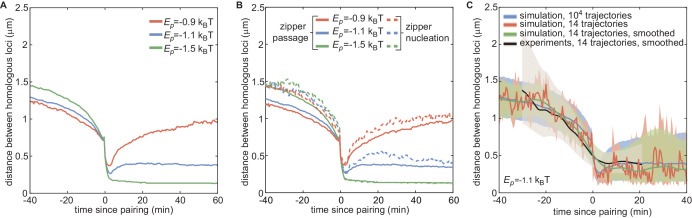
Impact of $E_{p}$ and small statistics on individual pairing dynamics. (**A**) Median pairing dynamics obtained from individual pairing trajectories detected during our simulations (colored lines) for ρ= 80% and different values of $E_{p}$. Traces are centered at the time of pairing (time = 0) defined as the time point where the inter-homolog distance decreases below 0.65 µm for at least 4 min. While the main differences lie in the post-pairing period, with stronger $E_{p}$ leading to a smaller distance between paired homologous loci, weak changes in the pre-pairing dynamics can be observed with stronger $E_{p}$ leading to faster decay in the inter-homolog distance. (**B**) For the same trajectories obtained in (**A**), we clustered the individual pairing trajectories into two categories. First, trajectories corresponding to primary pairing events (dashed lines), that is when no other loci in a ±200kbp window become paired earlier. Second, all the other trajectories (full lines), that is when at least another locus in the neighborhood becomes paired earlier. When $E_{p}$ is strong enough to maintain pairing, the first category corresponds to the nucleations of zippers while the second to the passage of a zipper. As expected, the pre-pairing dynamics of primary events (dashed lines) do not depend on $E_{p}$, reflecting the diffusion origin of these events. On the contrary, the passage events exhibit a faster pre-pairing dynamics for stronger energies, suggesting a more efficient zippering. (**C**) Impact of small statistics and smoothing on the median pairing dynamics. For *ρ *= 80% and $E_{p}=-1.1kT$, we randomly picked n = 14 trajectories (as in the experimental set) from our 10,0000 simulated trajectories, computed the median (orange line) and interquartile range (pink area) and smoothed them (green line and light gray area) with the same parameters used for the experimental curves (black line and gray area, see Figure 5—figure supplement 1C). In the pre-pairing dynamics, the small statistics (n = 14) and smoothing may slightly perturb the overall shape of the predicted median dynamics while still largely remaining within the interquartile range found using the full statistics (blue line and blue area).

**Figure 5—video 1.** Representative distance trajectory of two loci denoted as ‘pairing’ (plotted in the black experimental trace in Figure 5A) showing a rapid transition from large distances at earlier time points to smaller distances at later time points. The trajectory is shown alongside movies of the nucleus and gastrulating embryo from which the distances were calculated to help visualize the speed at which this pairing occurs. Image stacks were taken roughly every 30 s and max-projected for 2D viewing.

In contrast to the initial pairing dynamics, varying model parameter values had a clear effect on the distance dynamics that followed the pairing event (Figure 5—figure supplement 2A). Specifically, simulations with a weak $E_{p}$ led to a slow increase in inter-homolog distances as time progressed (Figure 5C, red), consistent with unstable pairing events. Conversely, simulations with a strong $E_{p}$ were associated with tight pairing of homologous loci following the pairing event, with inter-homolog distances stably maintained around 130 nm, close to the spatial resolution of the model (Figure 5D, green). Notably, the values of ρ and $E_{p}$ that best fit the averaged temporal evolution of the fraction of paired loci over development (Figures 4 and 5B) all led to similar predictions for the median inter-homolog distance dynamics associated with pairing events. These traces converged to a stable long-term median inter-homolog distance of ~0.5 µm (Figure 5E,F), which is nearly identical to the experimentally determined distance of ~0.44 µm between homologous loci in stably paired nuclei (compare the colored and black lines in Figure 5E,F). Our results thus suggest that the slow dynamics of the pairing probability observed during development (Figure 4) and the dynamics of inter-homolog distance after a pairing event (Figure 5) are strongly correlated.

We then compared our simulated traces to experimental observations of pairing events in nuclei of live embryos. Among the movies that we monitored, we captured 14 pairing events matching the criteria of initial large inter-homolog distances that drop below 0.65 µm for at least 4 min (Figure 5A; Figure 5—figure supplement 1; Figure 5—video 1). We aligned each of these pairing events using the same approach as with the simulated data described above and calculated the smoothed median dynamics of inter-homolog distances around the pairing event (Figure 5C–F, black lines, see also Figure 5—figure supplement 1C). The pre-pairing dynamics were fully compatible with model predictions, with a rapid decrease in inter-homolog distances over 10–20 min (Figure 5C–F, see also Figure 5—figure supplement 2C). Furthermore, the experimental post-pairing dynamics in inter-homolog distance were closely recapitulated (Figure 5F) by the predictions made using parameters that best fit the pairing probability over developmental time (Figure 5B). In sum, simulations of chromosomal behavior based on a button model with a defined set of parameters quantitatively recapitulate experimental observations of pairing events at individual loci, of stably paired homologs following a pairing event, and of the global progression of pairing dynamics over developmental time.

## Discussion

Since its discovery by Nettie Stevens over 100 years ago (Stevens, 1908), somatic homolog pairing has represented a fascinating puzzle for geneticists and cell biologists alike. The dissection of the molecular origins of somatic pairing presents a tractable case study to further our understanding of the 3D organization of chromosomes and the functional consequences of interactions among otherwise distant DNA loci. However, despite decades of research, the molecular mechanisms underlying somatic homolog pairing have remained elusive (Joyce et al., 2016). In this paper, we augmented the emerging button-based cartoon model of somatic homolog pairing by turning it into a precise theoretical model that makes quantitative and testable predictions of pairing dynamics as a function of the density of buttons throughout the chromosome and the specific interaction energy between buttons.

To assess the feasibility of this button model, we used it to predict chromosomal dynamics and then tested those predictions experimentally by tracking pairing dynamics at individual chromosomal loci in living embryos. Simulations predicted rapid transitions from unpaired to paired states resulting from a ‘zippering’ effect across the chromosomes where buttons that become paired via random encounters promote and stabilize pairing of adjacent buttons (Figure 2B). The model predicts that the spread of pairing from button to button along the length of the chromosome ultimately leads to the formation of paired homologous chromosomes that remained associated throughout the remainder of the simulation. This process gives rise to significant large-scale correlations between the pairing probabilities of distant loci, spreading over large genomic distances (~Mbp) as global pairing progresses during development (Figure 2—figure supplement 3).

The notion of zippering was previously proposed in a classical model of somatic pairing by Ed Lewis (Duncan, 2002) although, in his model, pairing initiates exclusively from the centromeres and propagates out toward the telomeres. In contrast, our data shows that pairing initiates randomly at multiple chromosomal positions. In this way, our model supports a prior study that used DNA-FISH on fixed embryos to demonstrate that pairing initiates at independent loci along the chromosome (Fung et al., 1998), and is consistent with polymer modeling that also suggests zippering as a possible mechanism for meiotic pairing (Marshall and Fung, 2016). While our experimental validation of the button model is currently limited to the tracking of a single pair of homologous loci at a time, the simultaneous live imaging of several loci would enable a more complete test of the collective, large-scale dynamics emerging from the predicted zippering process. Recent progress in the labeling of multiple loci may make this challenge possible in the coming years (Chen et al., 2018).

In tracking pairing dynamics through early development in living embryos, we found quantitative agreement with the button model predictions: the transition from an unpaired to a paired state is a rapid event that occurs in just a few minutes (Figure 5A), and paired chromosomal loci remain stably paired over the observation time of our experiments, up to 45 min (data not shown). Overall, the close quantitative agreement between observation and theory validates the button model as a mechanism that supports the initiation and maintenance of somatic homolog pairing. Furthermore, our measurements constrain the range of possible values of the button density and interaction energy (Figure 4C).

Two caveats may be considered in interpreting our analysis. First, our method of tracking homologous loci in living embryos relies on visualizing nascent RNAs generated from transgenes (Materials and methods) rather than direct observations of DNA or DNA-binding proteins. While nascent RNAs provide a robust and convenient signal for the position of the underlying DNA (Lim et al., 2018; Chen et al., 2018), the method limits us to examining the behavior of transcriptionally active loci, which could behave differently from silent chromatin. In addition, our analysis could overestimate inter-homolog distances in paired nuclei if, for example, nascent RNA molecules from separate chromosomes are prevented from intermixing (Fay and Anderson, 2018). Second, our simulations do not account for complex behaviors of the genome that take place during development and that may also influence pairing dynamics and stability, including cell-cycle progression and mitosis (Foe, 1989), establishment of chromatin states and associated nuclear compartments (Sexton et al., 2012; Yuan and O'Farrell, 2016; Hug et al., 2017; Ogiyama et al., 2018), and additional nuclear organelles such as the histone locus body (White et al., 2011; Liu et al., 2006). Further testing and refinement of our theoretical and molecular understanding of somatic homolog pairing will require new approaches to incorporate the potential influences of these genomic behaviors in a developmental context.

A previous analysis of pairing and transvection in living embryos focused on the blastoderm phase, coinciding with the earliest developmental time points in our analysis, and found that inter-homolog interactions were generally unstable at that time (Lim et al., 2018). Thus, the embryo appears to transition from an early state where pairing is not stable prior to cellularization to one that supports stable pairing at later time points of development. Prior studies have postulated changes in cell-cycle dynamics (Fung et al., 1998; Gemkow et al., 1998), chromatin states (Bateman and Wu, 2008), or proteins that promote or antagonize pairing (Joyce et al., 2012; Bateman et al., 2012a; Hartl et al., 2008; Rowley et al., 2019) as potentially mediating a shift to stable pairing during the maternal-to-zygotic transition that occurs during blastoderm cellularization. Our data suggest that these changes mediate their effect on pairing by directly or effectively modulating button activity.

What is the molecular nature of the buttons? Prior studies based on Hi-C methods reported that the *Drosophila* genome contains small (a few kbps) distinct regions or peaks of tight pairing between homologs distributed with a typical density of 60–70% throughout the chromosome, which could represent pairing buttons (AlHaj Abed et al., 2019; Erceg et al., 2019; Rowley et al., 2019). Given such a button density and our experimental observations, our model predicts that a specific interaction energy between buttons would be ~1–2 k_B_T (Figure 4C), a value consistent with both typical protein–protein interactions (Phillips et al., 2012) and with electrostatic interactions between homologous DNA duplexes (Kornyshev and Leikin, 2001; Gladyshev and Kleckner, 2014).

Recently, an analysis of ectopically induced pairing in vivo by Viets et al., 2019 found that relatively large chromosomal segments (~100 kbp) are required to promote pairing, consistent with our model prediction that a region must contain enough ‘small’ buttons (or tight-pairing regions) at a given interaction strength to become paired (Figure 4C). Moreover, two studies independently found enrichment for DNA-binding architectural and insulator proteins in tight-pairing regions (AlHaj Abed et al., 2019; Rowley et al., 2019), suggesting a potential role for these proteins in button function. In support of this view, Viets et al., 2019 observed that genomic regions amenable to pairing are enriched in clusters of insulator proteins, and previous works on the incorporation of insulator sequences into transgenes showed that these sequences can stabilize pairing and transvection (Lim et al., 2018; Fujioka et al., 2016; Piwko et al., 2019). Notably, our analysis revealed a requirement for some degree of specificity between homologous buttons (Figure 2—figure supplement 4A,B), since simulations of non-specific interactions between buttons did not result in robust pairing (Figure 2—figure supplement 2E). Perhaps a ‘code’ of interactions between unique combinations of insulators and architectural proteins (AlHaj Abed et al., 2019; Rowley et al., 2019) conveys the necessary specificity between homologous buttons for efficient pairing (Figure 2—figure supplement 4A,C). Another complementary possibility is that buttons may form large self-interacting pairing units or specific microcompartments along the genome (Figure 2—figure supplement 4D,E), potentially overlapping with the segmentation of the genome into TADs (Viets et al., 2019).

While somatic homolog pairing is widespread in *Drosophila* and other Dipterans, it is curious that pairing of homologous sequences is rare in the somatic cells of other diploid species. It is possible that the sequences and proteins that underlie buttons are unique to Dipterans and are not present on the chromosomes of other species, perhaps due to the diversity of architectural proteins carried in the *Drosophila* genome (Cubeñas-Potts and Corces, 2015). Alternatively, chromosomes of other species may have the capacity to pair through encoded buttons, but are prevented from doing so by the functions of proteins that antagonize pairing, such as the condensin II complex (Hartl et al., 2008; Joyce et al., 2012; Rowley et al., 2019). However, most other diploid species do show a capacity to pair homologous chromosomes during the early stages of meiosis, and polymer models similar to ours have been proposed as potential mechanisms for meiotic pairing (Marshall and Fung, 2016; Penfold et al., 2012; Nicodemi et al., 2008b). While it is possible that meiotic pairing could be mediated via buttons similar to those postulated here (Marshall and Fung, 2016), important differences appear to exist in the progression of meiotic pairing relative to somatic pairing, such as the highly dynamic and unstable associations between homologous loci (Ding et al., 2004) and rapid meiotic prophase chromosome movements (Lee et al., 2012) that have been observed in yeast, as well as unique chromosomal regions called pairing centers in *Caenorhabditis elegans* (MacQueen et al., 2005; Phillips et al., 2005). Therefore, multiple molecular mechanisms may accomplish the goal of aligning homologous chromosomes in different cellular contexts.

Importantly, our biophysical model of the otherwise cartoon-like button model coupled with quantitative live-cell imaging of pairing dynamics establishes a foundational framework for uncovering the parameters of button density and binding energy underlying somatic homolog pairing. In the future, we anticipate that our model will be instrumental in identifying and characterizing candidate button loci and in determining how these parameters are modulated in the mutant backgrounds that affect pairing (Bateman et al., 2012b; Joyce et al., 2012; Hartl et al., 2008; Gemkow et al., 1998). For example, titration of candidate pairing factors such as specific insulator proteins may challenge the role of the strength of interactions in maintaining a proper global level of pairing as predicted by the button model (Figure 2D, left; Figure 2—figure supplement 2A). Deletion of buttons at specific loci may also help dissect the role of button density (Figure 2D, center) and the propagation of local perturbations to distal loci (Figure 2—figure supplement 3B). Thus, our study significantly advances our understanding of the century-old mystery of somatic homolog pairing and provides a theory-guided path for uncovering its molecular underpinnings.

## Materials and methods

### The homologous button model

We modeled two pairs of homologous chromosome arms as semi-flexible self-avoiding polymers. Each chromosome consists of N = 3200 beads, with each bead containing 10 kbp and being of size *b* nm. The four polymers moved on a face-centered-cubic lattice of size *L_x_ x L_y_ x L_z_* under periodic boundary conditions to account for confinement by other chromosomes. Previously, we showed that TAD and compartment formation may be quantitatively explained by epigenetic-driven interactions between loci sharing the same local chromatin state (Jost et al., 2014; Ghosh and Jost, 2018). However, such weak interactions cannot lead to global homologous pairing (Pal et al., 2019). Here, to simplify our model, we neglect these types of interactions (whose effects are mainly at the TAD scale) to focus on the effect of homolog-specific interactions. However, we do consider HP1-mediated interactions between (peri)centromeric regions that are thought to impact the global large-scale organization inside nuclei (Figure 2—figure supplement 2D; Strom et al., 2017).

Homologous pairing was modeled as contact interactions between some homologous monomers, the so-called buttons (Figure 2A). For each pair of homologous chromosomes, positions along the genome were randomly selected as buttons with a probability ρ. Each 10-kbp bead *i* of chromosome *chr* is therefore characterized by a state *p_chr,i_* with *p_chr,i_ = 1* if it is a button (= 0 otherwise) and *p_chr,i_ = p_chr’,i_* = 1 if *chr* and *chr’* are homologous. In addition, the first 1000 monomers of each chromosome were modeled as self-attracting centromeric and pericentromeric regions, the rest as neutral euchromatic regions. The energy of a given configuration was given by

(S1) $$H=\sum_{chr}^{}k\sum_{i}^{}\left(1-cos\theta_{i,chr}\right)+\sum_{chr,chr^{\prime },i,j}^{}\left[E_{p}\delta_{chr,i;chr^{\prime },j}\Delta_{chr,i;chr^{\prime },j}p_{chr,i}+E_{c}\delta_{chr,i;chr^{\prime },j}C_{chr,i}C_{chr^{\prime },j}\right],$$

where *k* is the bending rigidity, *θ_i,chr_* is the angle between the bond vectors *i* and *i+1* of chromosome *chr*, $\delta_{chr,i;chr',j}=1$ if beads *i* from chromosome *chr* and *j* from *chr’* occupy nearest-neighbor sites (= 0 otherwise), $\Delta_{chr,i;chr',j}$ = 1 if *i = j* and *chr* and *chr’* are homologous (= 0 otherwise), $C_{chr,i}$ = 1 if bead *i* of *chr* is a (peri-)centromeric region, $E_{p}$ < 0 is the contact energy between homologous buttons, $E_{c}$ < 0 is the contact energy between centromeric beads.

The dynamics of the chains followed a simple kinetic Monte-Carlo scheme with local moves using a Metropolis criterion applied to *H*. The values of *k* (*=1.5kT*), *b* (*=105* nm), $E_{c}$(*=−0.1kT*), *L_x _= L_y_* (*=2* µm), and *L_z_* (*=4* µm) were fixed using the coarse-graining and time-mapping strategies developed in Ghosh and Jost, 2018 for a 10 nm fiber model and a volumic density = 0.009 bp/nm^3^ typical of *Drosophila* nuclei. For every set of remaining parameters (the button density ρ and the strength of pairing interaction *E_p_*), 250 independent trajectories were simulated starting from compact, knot-free, Rabl-like initial configurations (Dernburg et al., 1996): all centromeric regions were localized at random positions at the bottom of the simulation box, the rest of the chain being confined into a cylinder of diameter ~600 nm and height ~2 µm pointing toward the top of the box (see examples in Figure 2C and Figure 2—video 1 and 2). The distance between the centers of mass of homologous chromosomes, noted as *d_i_*, typically varied between 0.5 µm and 3 µm. Each trajectory represented ~10 h of real time. To model the developmental pairing dynamics, we ran simulations in which *d_i_* was sampled from the distribution inferred from chromosome painting experiments (Figure 2—figure supplement 2).

To constrain model parameters, we compared the measured pairing dynamics (Figure 4B) to the model prediction. Specifically, for each parameter set, we computed a chi^2^-score between the predicted dynamics and experimental time points

(S2) $$chi^{2}=\left(1/5\right)\sum_{t=3h}^{5.5h}\left[{\left(P_{pred}\left(t\right)-P_{exp,38F}\left(t\right)\right)}^{2}/\left(2\sigma_{exp,38F}^{2}\left(t\right)\right)+{\left(P_{pred}\left(t\right)-P_{exp,53F}\left(t\right)\right)}^{2}/\left(2\sigma_{exp,53F}^{2}\left(t\right)\right)\right],$$

with $P_{pred}(t)$ the predicted dynamics at developmental time t, $P_{exp,38F}(t)$ and $P_{exp,53F}(t)$ the experimental average dynamics for loci 38F and 53F at time t, respectively, and $\sigma_{exp,38F}^{}(t)$ and $\sigma_{exp,53F}^{}(t)$ their corresponding standard deviations at time t.

### DNA constructs and fly lines

Flies expressing a nuclear MCP-NLS-mCherry under the control of the nanos promoter were previously described (Bothma et al., 2018). To create flies expressing PCP-NoNLS-GFP, the plasmid pCASPER4-pNOS-eGFP-PCP-ɑTub3′UTR was constructed by replacing the MCP coding region of pCASPER4-pNOS-NoNLS-eGFP-MCP-ɑTub3′UTR (Garcia et al., 2013) with the coding region of PCP (Larson et al., 2011). Transgenic lines were established via standard P-element transgenesis (Spradling and Rubin, 1982). To create flies expressing MS2 or PP7 loops under the control of UAS, we started from plasmids piB-hbP2-P2P-lacZ-MS2-24x-αTub3′UTR (Garcia et al., 2013) and piB-hbP2-P2P-lacZ-PP7-24x-αTub3′UTR, the latter of which was created by replacing the MS2 sequence of the former with the PP7 stem loop sequence (Larson et al., 2011). The *hunchback* P2P promoter was removed from these plasmids and replaced by 10 copies of the UAS upstream activator sequences (Brand and Perrimon, 1993) and the *Drosophila* Synthetic Core Promoter (DSCP) (Pfeiffer et al., 2010). Recombinase-mediated cassette exchange (Bateman et al., 2006) was then used to place each construct at two landing sites in polytene positions 38F and 53F (Bateman and Wu, 2008; Bateman et al., 2012a). Flies carrying the GAL4 driver *nullo-GAL4,* which drives expression in all somatic cells during the cellular blastoderm stage of cell cycle 14, were a gift from Jason Palladino and Barbara Mellone. Flies carrying the GAL4 driver *R38A04-GAL4*, which drives expression in epidermal cells in germband-extended embryos (Jenett et al., 2012), were acquired from the Bloomington *Drosophila* Stock Center. Finally, the interlaced MS2 and PP7 loops under the control of the *hunchback* P2 enhancer and promoter (P2P-MS2/PP7-lacZ) were based on a previously described sequence (Wu et al., 2014).

To create embryos for analysis of pairing, mothers of genotype *10XUAS-DSCP-MS2; MCP-mCherry-NLS, PCP-GFP* were crossed to males of genotype *nullo-GAL4, 10XUAS-DSCP-PP7.* The resulting embryos are loaded with MCP-mCherry-NLS and PCP-GFP proteins due to maternal expression via the nanos promoter, and zygotic expression of *nullo-GAL4* drives transcription of MS2 and PP7 loops in all somatic cells starting approximately 30 min into cell cycle 14 (cellular blastoderm). For pairing analysis, both MS2 and PP7 transgenes were in the same genomic location, either position 38F or 53F, whereas for the negative control, MS2 loops were located at 38F and PP7 loops were located at 53F. To visualize pairing at later times in development, the mothers indicated above were instead crossed to males of genotype *10XUAS-DSCP-PP7; R38A04-GAL4*, where both MS2 and PP7 loops were located at position 38F. Finally, to visualize MS2 and PP7 loops derived from the same genomic location, mothers of genotype *MCP-mCherry-NLS, PCP-GFP* were crossed to P2P-MS2/PP7-lacZ located at position 38F.

### Embryo preparation and image acquisition

Embryos were collected at 25°C on apple juice plates and prepared for imaging as previously described (Garcia et al., 2013). Mounted embryos were imaged using a Leica SP8 confocal microscope, with fluorescence from mCherry and eGFP collected sequentially to minimize channel crosstalk. For each movie, the imaging window was 54.3 × 54.3 µm at a resolution of 768 × 768 pixels, with slices in each z-series separated by 0.4 µm. Z-stacks were collected through either 10 or 12 µm in the z plane (26 or 31 images per stack), resulting in a time resolution of approximately 27 or 31 s per stack using a scanning speed of 800 Hz and a bidirectional scan head with no averaging. For the pairing data in Figure 3, the imaging window was centered laterally for embryos that were pre-gastrulation; for post-gastrulation embryos, the imaging window centered on a dorsal view of the embryonic head region covering mitotic domains 18 and 20 (Foe, 1989), which shows minimal movements during gastrulation and germ band extension relative to other regions of the embryo. We compared pairing levels in these cells at 6 hr of development to that of cells in a posterior abdominal segment at the same time point and found them to be nearly identical (75.0% paired, n = 16 for anterior cells vs. 73.7%, n = 19 in posterior cells according to the definition of pairing in Figure 3E), confirming that cells from different regions of the embryo are roughly equivalent for pairing dynamics at this stage. For positive-control embryos with interlaced PP7 and MS2 loops driven by the *hunchback* promoter, embryos were imaged during cell cycle 13 and early cell cycle 14, and the imaging window was positioned laterally as previously described (Garcia et al., 2013). To assess pairing in late-stage embryos using the *R38A04-GAL4* driver, embryos were aged to approximately 11–12 hr and the imaging window was positioned laterally over an abdominal segment. For the developmental time course movies in Figure 4, imaging centered on mitotic domains 18 and 20 when these cells were in interphase. During time points when these domains were undergoing mitosis, an adjacent mitotic domain in interphase was imaged.

### Image analysis

All images were first run through the ImageJ plug-in Trainable Weka Segmentation (Arganda-Carreras et al., 2017) and filtered with custom classifiers to generate two separate channels of 3D segmented images that isolated fluorescent spots. These segmented spots were then fitted to a Gaussian with a nonlinear least squares regression to find the 2D center. Image z-stacks were then searched for any spots tracked for three or more contiguous z-slices and the r est were discarded. Additional manual curation was employed to confirm the accuracy of segmented images and to add any spots that were missed. An initial estimate of the center of each spot was set based on the z-slice in which the spot had the greatest maximum intensity within a predefined radius from its 2D center. These initial estimates were then used to seed a 3D Gaussian fit for each spot, the center of which was used for all distance calculation. This granted us not only sub-pixel resolution in x-y but also sub-z-slice resolution, allowing for more precision in the z coordinate, which would otherwise be limited by the 0.4 µm spacing between consecutive stacked images created by confocal imaging.

Raw image z-stacks for each time frame were also maximum projected in the channel containing nuclearly localized MCP-mCherry to create 2D maps of all the nuclei in frame. These nuclear projections were then segmented and tracked in Matlab, followed by manual curation to ensure that each nucleus was consistently followed. One tracked particle lineage from each channel was then assigned a distinct nucleus based on its proximity to that nucleus in the 2D map and the particles in each channel were considered homologous chromosomes of one another. Since absolute coordinates of assigned particles were not possible to obtain due to cellular rotation and motion, all distance calculations were done with the relative coordinates of each locus from its homolog; any cellular rotation or motion was assumed to be conserved between loci in the same cell.

For the data in Figure 3, we qualitatively scored each nucleus based on the measured distances between red and green signals over the time that the signals were observed: ‘paired’ nuclei showed small distances and little variation over time and ‘unpaired’ nuclei showed larger distances and greater variation over time. Nuclei that showed a transition from large distances and variation at earlier time points to smaller distances and variation at later time points were scored as ‘pairing’ traces and were not included in Figure 3 (see Figure 5). In assessing the stability of the paired state, we included both ‘paired’ (n = 25) and ‘pairing’ (n = 13) nuclei from three embryos in the total number of nuclei (n = 38) assessed. In this analysis, we conservatively only included the observation time of ‘paired’ nuclei (> 8 hr of observation with no transition back to the unpaired state), although ‘pairing’ nuclei also remained in the paired state throughout the remaining observation time once they became paired.

In some traces, signal is temporarily lost, which could be due to either a loss of fluorescence of the MS2 or PP7 reporters caused by transcriptional bursting or due to one or both loci moving out of our imaging window. For paired loci, we randomly sampled six traces and found that only two had any missing frames, with the missing events due solely to loss of transcription (three lost frames out of 339 total tracked frames in the sampling). Therefore, missing frames do not significantly impact our measurements of paired loci. In the case of unpaired loci, where the relative movement of homologous loci is less restricted, there is a greater risk of systematically underestimating the mean distance between signals if missing frames are caused by at least one homolog moving out of the field of view. To investigate this, we randomly sampled six unpaired loci traces and found that four of the six traces had missing frames with at least one signal outside of the imaging window (54 outside-of-window frames out of 587 total tracked frames in the sampling). To probe the possible impact on the mean distance of these traces, we assumed that the distance between homologs in all the missing frames of the six sampled traces was 5 µm, corresponding to the average nucleus diameter. While it is unlikely that all our missing frames contained loci that were 5 µm apart, this approach gives us an upper bound of the possible impact of missing frames. We found a rather modest effect with an increase of the mean distance of ~12.5% (from 2.4 µm to 2.7 µm) that is unlikely to alter any of our conclusions.

To align the traces presented in Figure 5 based on a time point when the loci become paired, we manually aligned all traces that had been qualitatively assessed as ‘pairing’ traces according to several values of threshold distance and consecutive frames below that threshold. We then optimized this exploration for values that provided qualitatively good alignment of traces but that excluded as few traces as possible in order to maximize the data available for analysis. The same criteria were applied to identify and align pairing traces from simulations.

All image analysis was done using custom scripts in Matlab 2019b unless otherwise stated. These scripts can be found at https://github.com/GarciaLab/mRNADynamics/ (Garcia Lab, 2021a, copy archived at swh:1:rev:a1b5c591656cae816ed6fc4a4e447c3bd375959c, Garcia Lab, 2021b).

### Chromosome painting

Embryos of genotype *w^1118^* were aged to 2–3 hr after embryo deposition, fixed, and subjected to DNA-FISH using 400 pmol of Oligopaint probes (Beliveau et al., 2012) targeting 2L and 2R (200 pmol of each probe; Rosin et al., 2018) as previously described (Bateman and Wu, 2008). Hybridized embryos were mounted in Vectashield mounting medium with DAPI (Vector Laboratories), and three-dimensional images were collected using a Leica SP8 confocal microscope. To establish initial inter-homolog distances, an image from an embryo in early interphase 14 (as judged by nuclear elongation [Fung et al., 1998]) and with high signal-to-noise ratio was analyzed using the TANGO image analysis plug-in for ImageJ (Ollion et al., 2013; Ollion et al., 2015; Belevich et al., 2016). After segmentation and assignment of each painted territory to a parent nucleus, distances between territories were measured from centroid to centroid in 3D. Since homologous chromosomes are labeled with the same color, when territories produce a continuous region of fluorescence, a distance of zero was assigned. A total of 48 nuclei were analyzed for each of 2L and 2R.

## Data Availability

Modeling code is available at: https://github.com/physical-biology-of-chromatin/Homologous_pairing (copy archived at https://archive.softwareheritage.org/swh:1:rev:09c00ff8e63d6fbe812660771fd2d22df277aa1a). Custom Matlab 2019b a image analysis scripts can be found at: https://github.com/GarciaLab/mRNADynamics/ (copy archived at https://archive.softwareheritage.org/swh:1:rev:a1b5c591656cae816ed6fc4a4e447c3bd375959c). Raw figure files of relevant plots are available at: https://doi.org/10.5281/zenodo.5063001. Samples of generated data used in this study are included in the manuscript and in supporting files available at: https://doi.org/10.5061/dryad.3j9kd51j5. The following datasets were generated: ChildMBBatemanJRJahangiriAReimerALammersNCSabouniNVillamarinDMcKenzie-SmithGCJohnsonJEJostDGarciaHG2021Live imaging and biophysical modeling support a button-based mechanism of somatic homolog pairing in DrosophilaDryad Digital Repository10.5061/dryad.3j9kd51j5PMC829484734100718 ChildMBBatemanJRJahangiriAReimerALammersNCSabouniNVillamarinDMcKenzie-SmithGCJohnsonJEJostDGarciaHG2021Live imaging and biophysical modeling support a button-based mechanism of somatic homolog pairing in DrosophilaZenodo10.5281/zenodo.5063001PMC829484734100718

## Additional files

Transparent reporting form
